# Supplementary material for: Facet-Dependent SERS Activity of Co3O4
Source: Int J Mol Sci. 2022 Dec 14;23(24):15930. doi: 10.3390/ijms232415930 (PMC9788474; doi:10.3390/ijms232415930)
Supplement: Supplementary file 1 [file ijms-23-15930-s001.zip › ijms-2090231-supplementary.pdf]

Supporting information for
Facet dependent SERS activity of Co_3O_4

Yibo Feng, Jiaxing Wang, Jixiang Hou, Xu Zhang, Yuhang Gao* and Kaiwen Wang*

Beijing Key Laboratory and Institute of Microstructure and Property of Advanced Materials, Faculty of Materials and Manufacturing, Beijing University of Technology, Beijing 100124, China

* Correspondence: yuhanggao163@163.com (Y.G.); 18811419320@163.com (K.W.)

Table of contents

1. **Supplementary figures**

   Figure S1. XRD pattern of Co_3O_4.

   Figure S2. XPS and VB-XPS spectra of Co_3O_4.

   Figure S3. UV spectra of Co_3O_4.

   Figure S4. Raman spectra of powders A and R6G.

   Figure S5. Fresh and three months later Raman spectrum of Co_3O_4+A.

2. **Supplementary original data**

   Data S1. INCAR for DFT calculation.

   Data S2. CONTCAR of Adenine adsorbed on C-100.

   Data S3. CONTCAR of Adenine adsorbed on C-111.

1. Supplementary figures

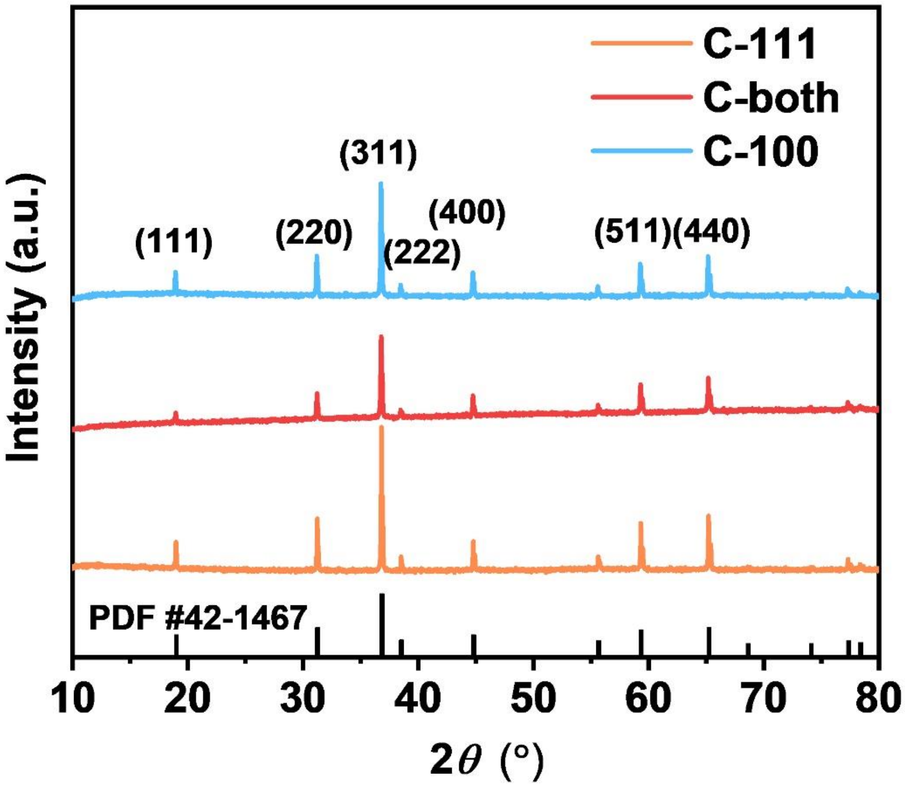

Figure S1. XRD patterns of C-100, C111 and C-both.

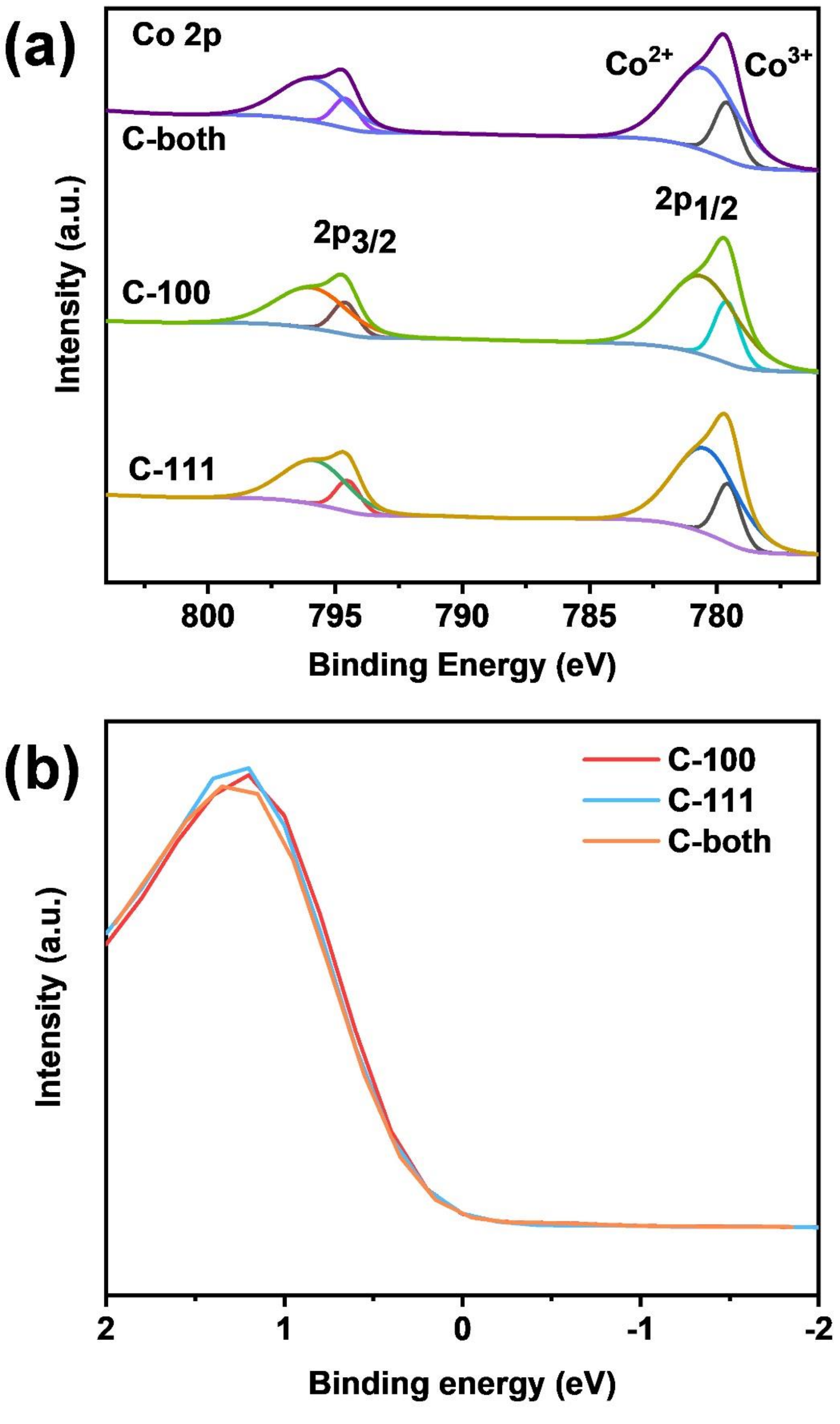

Figure S2. (**a**) XPS spectra of Co element. (**b**) VB-XPS spectra of C-100, C111 and C-both.

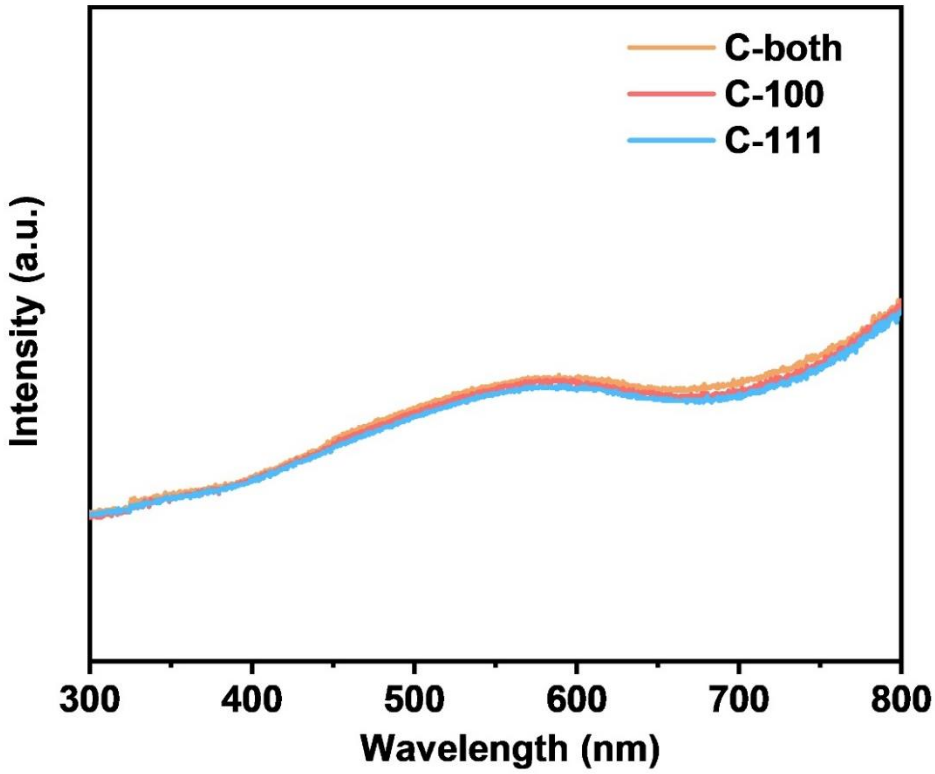

Figure S3. UV spectra of C-100, C111 and C-both.

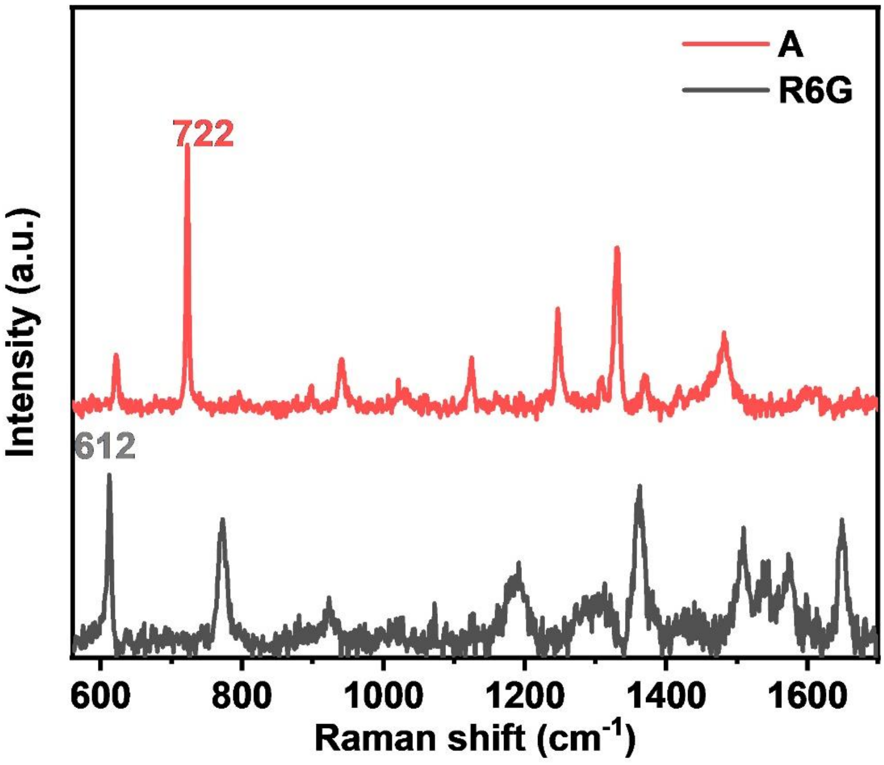

Figure S4. Raman spectra of powders A and R6G.

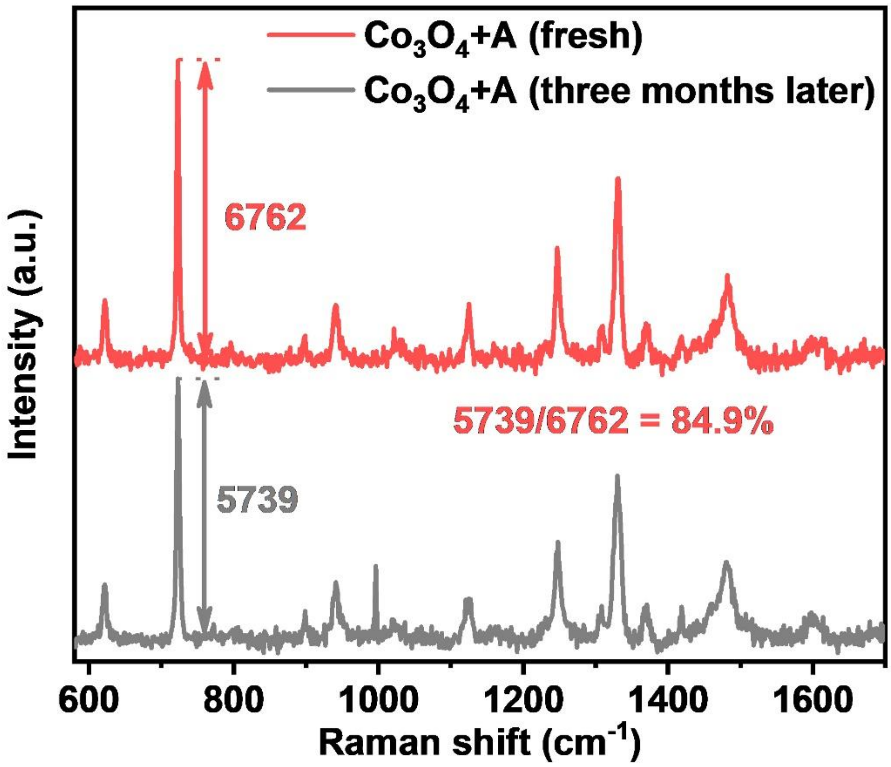

Figure S5. Fresh and three months later Raman spectrum of Co_3O_4+A.

2. Supplementary original data

```
INCAR

ENCUT=400
ISMEAR=0
SIGMA=0.1
EDIFF=0.1E-04
EDIFFG=-0.02
NSW=2000
IALGO=38
GGA=PE
IVDW=12
IBRION=2
ISPIN=2
LMAXMIX=4
AMIX=0.2
BMIX=0.0001    !most zero,but 0 will crash some versions
AMIX_MAG=0.8
BMIX_MAG=0.0001 !most zero,but 0 will crash some versions
NPAR=8
NELM=200
LCHARG=F
LWAVE=F
LREAL=Auto
ALGO =    Fast
LDAU=.TRUE.
LDAUTYPE=2
LDAUL= 2 -1 -1 -1 -1
LDAUU= 5.9 0.0    0 0 0
LDAUJ= 0.0 0.0 0 0 0
LDAUPRINT=2
```

Data S1. INCAR for DFT calculation.

CONTCAR-adenine adsorbed on Co_3O_4-(100)

```
   1.00000000000000
     22.5440998077000003    0.0000000000000000    0.0000000000000000
     -0.0194361919000000   22.5425922527999987    0.0000000000000000
      0.0000000000000000    0.0000000000000000   27.0573997497999983
   Co    O     N     C     H
   192   256   5     5     5
Selective dynamics
Direct
  0.2495999870000034  0.0000500000000017  0.0088300000000032   F   F   F
  0.1249200099999968  0.1250400039999988  0.1473300019999968   F   F   F
  0.0651099950000003  0.1250200039999996  0.0387800020000029   F   F   F
  0.1871600070000028  0.0000599999999977  0.1851199999999977   F   F   F
  0.9999300579999968  0.1867800050000028  0.1111199999999997   F   F   F
  0.1247771958227972  0.0629094520072574  0.2548775978296308   T   T   T
  0.1249699959999973  0.0000599999999977  0.0691500020000007   F   F   F
  0.9998270282754831  0.1250316521383751  0.2249073592017456   T   T   T
  0.9999200769999987  0.0633300029999972  0.1111399939999984   F   F   F
  0.1248134800234361  0.1870917637055121  0.2549052331644143   T   T   T
  0.1847599919999965  0.1250600139999989  0.0388100010000016   F   F   F
  0.0627399970000013  0.0000599999999977  0.1850499999999968   F   F   F
  0.4996199959999998  0.0000500000000017  0.0088599999999985   F   F   F
  0.3749400150000000  0.1250600139999989  0.1473599959999987   F   F   F
  0.3151300010000000  0.1250099989999995  0.0388100010000016   F   F   F
  0.4371500029999993  0.0000599999999977  0.1851299920000002   F   F   F
  0.2499100029999965  0.1868000150000029  0.1111199999999997   F   F   F
  0.3748528468859308  0.0629226716279548  0.2549421449259394   T   T   T
  0.3749899940000034  0.0000599999999977  0.0692099999999982   F   F   F
  0.2497795152097039  0.1250159008398106  0.2249262415173361   T   T   T
  0.2499400020000024  0.0633300029999972  0.1111699969999975   F   F   F
  0.3746712781612367  0.1870640519231213  0.2549425971581130   T   T   T
  0.4347499870000036  0.1250600139999989  0.0388300030000011   F   F   F
  0.3127300160000033  0.0000500000000017  0.1851199999999977   F   F   F
  0.7497999730000018  0.0000500000000017  0.0088500000000025   F   F   F
  0.6249500030000021  0.1250400039999988  0.1473599959999987   F   F   F
  0.5651199539999965  0.1250099989999995  0.0388000000000019   F   F   F
  0.6871599990000021  0.0000500000000017  0.1851199999999977   F   F   F
  0.4999099819999984  0.1868000150000029  0.1111399939999984   F   F   F
  0.6248195895176636  0.0630287252270569  0.2549099419837312   T   T   T
  0.6249700309999966  0.0000399999999985  0.0692299979999973   F   F   F
  0.4997370568178000  0.1249857386315532  0.2248861673392697   T   T   T
  0.4999299759999971  0.0633300029999972  0.1111799979999972   F   F   F
  0.6246576525013394  0.1872110145152209  0.2547486279833731   T   T   T
```

0.6847399819999964	0.1250600139999989	0.0388400000000004	F	F	F
0.5627200030000026	0.0000399999999985	0.1851199999999977	F	F	F
0.9998500290000010	0.0000399999999985	0.0088300000000032	F	F	F
0.8749399650000029	0.1250499989999980	0.1473400029999965	F	F	F
0.8151199750000018	0.1250099989999995	0.0387800020000029	F	F	F
0.9371399690000004	0.0000500000000017	0.1850499999999968	F	F	F
0.7499399109999985	0.1868000150000029	0.1111199999999997	F	F	F
0.8748244782749266	0.0630252321532168	0.2548898682922238	T	T	T
0.8748699810000033	0.0000100000000032	0.0691699999999997	F	F	F
0.7498282109392042	0.1250970389930748	0.2249176831927301	T	T	T
0.7499200049999999	0.0633200030000012	0.1111699969999975	F	F	F
0.8749667597862306	0.1872560118705485	0.2548696757927933	T	T	T
0.9347499949999971	0.1250200039999996	0.0388100010000016	F	F	F
0.8127200240000008	0.0000399999999985	0.1850999970000018	F	F	F
0.2497400080000034	0.2500199979999991	0.0087600000000023	F	F	F
0.1249400080000029	0.3750100279999984	0.1473599959999987	F	F	F
0.0651300030000002	0.3749900179999983	0.0387800020000029	F	F	F
0.1871400180000009	0.2500300239999973	0.1850800129999968	F	F	F
0.9999300019999993	0.4367600240000016	0.1111199999999997	F	F	F
0.1249359172463341	0.3128350756577380	0.2549177169265193	T	T	T
0.1249300010000027	0.2500100139999972	0.0691799969999991	F	F	F
0.0001445112848385	0.3750198149143750	0.2248862453381479	T	T	T
0.9999300039999994	0.3133100169999992	0.1111399939999984	F	F	F
0.1249410430569383	0.4371874591093609	0.2548966600908542	T	T	T
0.1847599979999970	0.3749900179999983	0.0387899979999986	F	F	F
0.0627299969999981	0.2500300239999973	0.1850699849999984	F	F	F
0.4997700210000033	0.2500199979999991	0.0088099999999969	F	F	F
0.3749500269999970	0.3750400229999968	0.1473499949999990	F	F	F
0.3151299869999988	0.3749900179999983	0.0387800020000029	F	F	F
0.4371399810000014	0.2500599970000010	0.1850999970000018	F	F	F
0.2499299810000011	0.4367600240000016	0.1111600050000021	F	F	F
0.3743065780387709	0.3132004684003254	0.2549060752904027	T	T	T
0.3749200119999969	0.2500400079999991	0.0691900019999991	F	F	F
0.2497739677831697	0.3751100767373761	0.2249218369313341	T	T	T
0.2499200079999966	0.3133200219999992	0.1111499949999981	F	F	F
0.3747768733717180	0.4374805970536056	0.2551715418648892	T	T	T
0.4347600239999991	0.3750200120000002	0.0388100010000016	F	F	F
0.3127099889999982	0.2500500129999992	0.1850900049999993	F	F	F
0.7499099799999982	0.2500199979999991	0.0088000000000008	F	F	F
0.6249600149999992	0.3750200120000002	0.1473799990000018	F	F	F
0.5651299740000013	0.3750000020000002	0.0388000000000019	F	F	F
0.6871500100000034	0.2500400079999991	0.1850800129999968	F	F	F
0.4999299690000001	0.4367700080000034	0.1111399939999984	F	F	F
0.6242445362959599	0.3135217714310260	0.2547098709765944	T	T	T

0.6249100160000012	0.2500500129999992	0.0692000029999988	F	F	F
0.4984381016127494	0.3753509322971748	0.2233306103529868	T	T	T
0.4999299790000009	0.3133300060000010	0.1111499949999981	F	F	F
0.6252890312246543	0.4374719449361651	0.2564809919507412	T	T	T
0.6847500189999991	0.3750200120000002	0.0388400000000004	F	F	F
0.5626999460000022	0.2500300239999973	0.1850900049999993	F	F	F
0.9997100360000033	0.2500199979999991	0.0087400000000031	F	F	F
0.8749700190000027	0.3750200120000002	0.1473799990000018	F	F	F
0.8151600130000034	0.3749800340000036	0.0387899979999986	F	F	F
0.9371499889999981	0.2500400079999991	0.1850699849999984	F	F	F
0.7499300229999974	0.4367600240000016	0.1111600050000021	F	F	F
0.8753288550717675	0.3129617415067233	0.2548419872035784	T	T	T
0.8749799460000034	0.2500300239999973	0.0691500020000007	F	F	F
0.7507610660741154	0.3752743687877101	0.2244586406509486	T	T	T
0.7499399240000031	0.3133100169999992	0.1111499949999981	F	F	F
0.8752927645099905	0.4372612342004639	0.2548645235175738	T	T	T
0.9347599569999971	0.3750400229999968	0.0388199969999974	F	F	F
0.8127400280000003	0.2500300239999973	0.1850699849999984	F	F	F
0.2497500200000005	0.5000000169999979	0.0088399999999993	F	F	F
0.1249500030000021	0.6250200420000027	0.1473799990000018	F	F	F
0.0651399999999995	0.6249900050000008	0.0388199969999974	F	F	F
0.1871499960000023	0.5000200269999979	0.1850999970000018	F	F	F
0.9999300570000003	0.6867700370000023	0.1111600050000021	F	F	F
0.1249653064925604	0.5628994878136371	0.2549037377600377	T	T	T
0.1249699930000006	0.5000000169999979	0.0691199979999979	F	F	F
0.0000215996731217	0.6250282576887382	0.2248602152861239	T	T	T
0.9999300500000032	0.5633100249999998	0.1111399939999984	F	F	F
0.1249438111953935	0.6871414650160431	0.2549038643422616	T	T	T
0.1847500060000016	0.6250500359999975	0.0388400000000004	F	F	F
0.0627399979999979	0.5000100430000032	0.1850499999999968	F	F	F
0.4998300190000009	0.5000300109999998	0.0088599999999985	F	F	F
0.3749600060000020	0.6250299840000011	0.1474100020000009	F	F	F
0.3151400160000009	0.6249900050000008	0.0388499969999998	F	F	F
0.4371499929999985	0.5000399950000016	0.1851100069999987	F	F	F
0.2499299850000014	0.6867600109999970	0.1111699969999975	F	F	F
0.3752284659217717	0.5629572734094742	0.2549556737312549	T	T	T
0.3749299979999989	0.4999899900000031	0.0691799969999991	F	F	F
0.2500542466241835	0.6250113940425500	0.2250429223770978	T	T	T
0.2499299990000026	0.5633000409999980	0.1111799979999972	F	F	F
0.3749948922867706	0.6871838541797253	0.2549248160771577	T	T	T
0.4347300009999984	0.6250299840000011	0.0388799999999989	F	F	F
0.3127099749999971	0.5000300109999998	0.1851100069999987	F	F	F
0.7498600150000030	0.5000100430000032	0.0088599999999985	F	F	F
0.6249900190000020	0.6250299840000011	0.1474200030000006	F	F	F

0.5651500290000016	0.6250000309999990	0.0388499969999998	F	F	F
0.6871700140000030	0.5000300109999998	0.1851100069999987	F	F	F
0.4999400060000028	0.6867799790000007	0.1111699969999975	F	F	F
0.6252800185045307	0.5631102686079089	0.2547415054571543	T	T	T
0.6249499700000030	0.5000200269999979	0.0691900019999991	F	F	F
0.5001479277461418	0.6250553053490842	0.2250198610747074	T	T	T
0.4999400000000023	0.5633200090000017	0.1111699969999975	F	F	F
0.6250573945005699	0.6873709177171827	0.2548461815041491	T	T	T
0.6847399809999999	0.6250500359999975	0.0388499969999998	F	F	F
0.5627099749999971	0.5000300109999998	0.1851100069999987	F	F	F
0.9999600060000020	0.5000200269999979	0.0088200000000001	F	F	F
0.8749500220000002	0.6250299840000011	0.1474000099999984	F	F	F
0.8151400329999987	0.6250100150000009	0.0388000000000019	F	F	F
0.9371500259999976	0.5000300109999998	0.1850599930000030	F	F	F
0.7499600279999967	0.6867700370000023	0.1111300009999994	F	F	F
0.8751677305213543	0.5630033061311979	0.2549347091447591	T	T	T
0.8748900479999975	0.5000200269999979	0.0691400010000010	F	F	F
0.7501513237936976	0.6250919566828335	0.2249490553297190	T	T	T
0.7499300710000014	0.5633100249999998	0.1111600050000021	F	F	F
0.8750745745250066	0.6872930622870471	0.2549096064379129	T	T	T
0.9347699599999970	0.6250100150000009	0.0388499969999998	F	F	F
0.8127400819999977	0.5000399950000016	0.1850999970000018	F	F	F
0.2497799949999973	0.7500500089999989	0.0088200000000001	F	F	F
0.1249499939999978	0.8750300129999999	0.1473899909999972	F	F	F
0.0651300030000002	0.8750500660000000	0.0388100010000016	F	F	F
0.1871400039999997	0.7500300410000023	0.1851199999999977	F	F	F
0.9999300349999984	0.9367900350000014	0.1111300009999994	F	F	F
0.1248742953029348	0.8128796751249286	0.2549311915432785	T	T	T
0.1249500010000020	0.7500400249999970	0.0692200009999979	F	F	F
0.9999607983730010	0.8750923349273710	0.2248927661887730	T	T	T
0.9999399949999983	0.8133100120000023	0.1111699969999975	F	F	F
0.1248808562551070	0.9371535058563784	0.2548946323024022	T	T	T
0.1847500009999976	0.8750399970000018	0.0388499969999998	F	F	F
0.0627199990000022	0.7500300410000023	0.1851100069999987	F	F	F
0.4997900119999983	0.7500400249999970	0.0088200000000001	F	F	F
0.3749499919999977	0.8750399970000018	0.1474100020000009	F	F	F
0.3151400240000015	0.8750200289999981	0.0388400000000004	F	F	F
0.4371700049999987	0.7500400249999970	0.1851199999999977	F	F	F
0.2499399969999985	0.9367900350000014	0.1111799979999972	F	F	F
0.3748376282239505	0.8129101883100081	0.2549440678282949	T	T	T
0.3749300019999993	0.7500400249999970	0.0692299979999973	F	F	F
0.2498517376521233	0.8750200748995056	0.2249298564424199	T	T	T
0.2499200070000001	0.8133100120000023	0.1111699969999975	F	F	F
0.3748513931587429	0.9371390211448662	0.2548832868775534	T	T	T

0.4347499799999994	0.8750399970000018	0.0388699989999992	F	F	F
0.3127099789999974	0.7500300410000023	0.1851199999999977	F	F	F
0.7497699560000015	0.7500100720000020	0.0087900010000013	F	F	F
0.6249600049999984	0.8750500660000000	0.1473799990000018	F	F	F
0.5651500499999997	0.8750200289999981	0.0388300030000011	F	F	F
0.6871799589999981	0.7500300410000023	0.1850800129999968	F	F	F
0.4999299759999971	0.9367700669999977	0.1111999999999966	F	F	F
0.6249662332873728	0.8131201687883485	0.2548624229778436	T	T	T
0.6249399650000029	0.7500400249999970	0.0692000029999988	F	F	F
0.4998854766809280	0.8750688913294833	0.2249686544255835	T	T	T
0.4999299690000001	0.8133100120000023	0.1111799979999972	F	F	F
0.6250184529947352	0.9373272033762622	0.2548596610832116	T	T	T
0.6847699679999977	0.8750399970000018	0.0388400000000004	F	F	F
0.5627299880000010	0.7500300410000023	0.1851199999999977	F	F	F
0.9995999890000036	0.7500400249999970	0.0087800000000016	F	F	F
0.8749599670000023	0.8750300129999999	0.1473599959999987	F	F	F
0.8151400619999976	0.8749899920000033	0.0388000000000019	F	F	F
0.9371799460000005	0.7500400249999970	0.1851199999999977	F	F	F
0.7499300300000016	0.9367599979999994	0.1111699969999975	F	F	F
0.8750200707815529	0.8130689983376596	0.2548729720792194	T	T	T
0.8750299370000008	0.7500100720000020	0.0691699999999997	F	F	F
0.7500047858642893	0.8750589679256612	0.2248794524230906	T	T	T
0.7499499910000011	0.8133000280000005	0.1111300009999994	F	F	F
0.8749721329638045	0.9373147655580830	0.2548360563963861	T	T	T
0.9347599720000019	0.8750500660000000	0.0388300030000011	F	F	F
0.8127499340000028	0.7500300410000023	0.1850800129999968	F	F	F
0.1249200060000035	0.0695400030000002	0.0372800009999992	F	F	F
0.2499200000000030	0.1920900140000015	0.1822600059999999	F	F	F
0.2499200060000035	0.0697000070000016	0.0370599979999966	F	F	F
0.1248999990000002	0.1923700130000014	0.1845499960000012	F	F	F
0.0576499989999988	0.2500800079999976	0.1126000029999972	F	F	F
0.1795593732262035	0.1250193178756428	0.2554824825658902	T	T	T
0.1249900050000008	0.1805899989999986	0.0372599989999998	F	F	F
0.2499899940000034	0.0580200039999994	0.1822800079999993	F	F	F
0.1922299989999985	0.2500100139999972	0.1126099949999997	F	F	F
0.0700505615214201	0.1249606774897741	0.2554107299443376	T	T	T
0.1922499980000012	0.1250000040000003	0.1096700009999978	F	F	F
0.0704006426141486	0.2499776253697423	0.2551595862264212	T	T	T
0.2499700119999986	0.1804000059999993	0.0370200019999984	F	F	F
0.1250000040000003	0.0577200050000002	0.1845499960000012	F	F	F
0.0576399990000027	0.1251000039999965	0.1096499989999984	F	F	F
0.1794157589135552	0.2499920098869235	0.2551872180455372	T	T	T
0.3748999920000031	0.0695099980000009	0.0373399989999967	F	F	F
0.4998999909999995	0.1920900140000015	0.1822600059999999	F	F	F

0.4998999760000018	0.0697000070000016	0.0370800000000031	F	F	F
0.3749000150000015	0.1924000079999999	0.1845699980000006	F	F	F
0.3076399919999986	0.2500900129999977	0.1126000029999972	F	F	F
0.4296180059484433	0.1251984258359385	0.2558764786090132	T	T	T
0.3749699879999966	0.1805800149999968	0.0372599989999998	F	F	F
0.4999800109999981	0.0580200039999994	0.1822999930000009	F	F	F
0.4422300080000028	0.2500199979999991	0.1126099949999997	F	F	F
0.3201376433272571	0.1251333177511532	0.2553345425815124	T	T	T
0.4422299849999973	0.1250200039999996	0.1096900029999972	F	F	F
0.3202985060833316	0.2500430102720057	0.2553686851310099	T	T	T
0.4999699999999976	0.1804100109999993	0.0370399999999975	F	F	F
0.3749899829999990	0.0576999999999970	0.1845799909999997	F	F	F
0.3076500040000028	0.1251000039999965	0.1096700009999978	F	F	F
0.4297091946389691	0.2504212361775950	0.2563808490243834	T	T	T
0.6249000039999970	0.0695000030000017	0.0373500000000035	F	F	F
0.7499200119999969	0.1920800090000014	0.1822499960000030	F	F	F
0.7498800640000027	0.0696899969999976	0.0370800000000031	F	F	F
0.6248999929999997	0.1924000079999999	0.1845499960000012	F	F	F
0.5576500390000021	0.2500900129999977	0.1126099949999997	F	F	F
0.6795003913353866	0.1254142679784022	0.2554825017778586	T	T	T
0.6249800009999973	0.1805899989999986	0.0372699999999995	F	F	F
0.7499899640000010	0.0580100040000033	0.1822900009999984	F	F	F
0.6922500379999974	0.2500199979999991	0.1126099949999997	F	F	F
0.5699773120870606	0.1250633978976571	0.2557836964921099	T	T	T
0.6922399899999974	0.1250200039999996	0.1096900029999972	F	F	F
0.5692473473594313	0.2499122985816654	0.2550940770738988	T	T	T
0.7499699859999964	0.1803700110000008	0.0370299989999978	F	F	F
0.6249999699999975	0.0576799999999977	0.1845699980000006	F	F	F
0.5576500330000016	0.1251100089999966	0.1096799930000003	F	F	F
0.6792298205968935	0.2500383820652038	0.2549327360446558	T	T	T
0.8748800500000016	0.0694900029999985	0.0372899979999985	F	F	F
0.9999100080000005	0.1920800090000014	0.1822299939999965	F	F	F
0.9998800509999981	0.0697000070000016	0.0370599979999966	F	F	F
0.8749099560000033	0.1924000079999999	0.1845400039999987	F	F	F
0.8076900780000003	0.2500900129999977	0.1125900019999975	F	F	F
0.9296060817785060	0.1251535753155317	0.2554240729925283	T	T	T
0.8749600040000018	0.1805800149999968	0.0372599989999998	F	F	F
0.9999799599999974	0.0580100040000033	0.1822299939999965	F	F	F
0.9422499659999986	0.2500100139999972	0.1126000029999972	F	F	F
0.8200987843929589	0.1251156804424293	0.2554678464772856	T	T	T
0.9422299430000010	0.1250200039999996	0.1096400059999993	F	F	F
0.8205023818721275	0.2501922918761080	0.2552551862634866	T	T	T
0.9999499720000031	0.1803400169999989	0.0369999999999990	F	F	F
0.8749900250000024	0.0576999999999970	0.1845400039999987	F	F	F

0.8076400109999966	0.1250900089999973	0.1096599999999981	F	F	F
0.9296321330303664	0.2500368020531696	0.2551441593258289	T	T	T
0.1249099970000032	0.3194700060000031	0.0372699999999995	F	F	F
0.2499100020000000	0.4420700330000003	0.1822600059999999	F	F	F
0.2499000039999970	0.3196300059999970	0.0370100009999987	F	F	F
0.1249099990000033	0.4423600370000003	0.1845499960000012	F	F	F
0.0576699969999979	0.5000699900000001	0.1125800000000012	F	F	F
0.1796357530617617	0.3750417557563780	0.2554776781232641	T	T	T
0.1249999990000035	0.4305000029999988	0.0372399970000004	F	F	F
0.2499700130000022	0.3080000069999969	0.1822600059999999	F	F	F
0.1922499860000002	0.4999800060000013	0.1126200049999966	F	F	F
0.0702960594722781	0.3749637042466319	0.2555121162532796	T	T	T
0.1922300179999965	0.3749800340000036	0.1096499989999984	F	F	F
0.0704398853416505	0.4999900265248350	0.2551136075931318	T	T	T
0.2499800020000009	0.4303300199999995	0.0370299989999978	F	F	F
0.1249800060000013	0.3076800300000002	0.1845600059999981	F	F	F
0.0576499989999988	0.3750599910000005	0.1096400059999993	F	F	F
0.1794505748087840	0.5000420313578159	0.2551476029264289	T	T	T
0.3749099950000030	0.3194800119999996	0.0372800009999992	F	F	F
0.4999099690000008	0.4420800179999986	0.1822600059999999	F	F	F
0.4999000039999970	0.3196300059999970	0.0370100009999987	F	F	F
0.3748999920000031	0.4423700210000021	0.1845799909999997	F	F	F
0.3076500080000031	0.5000500220000035	0.1126099949999997	F	F	F
0.4294553661429899	0.3751575433039991	0.2549347219828554	T	T	T
0.3749800160000021	0.4305300400000007	0.0372899979999985	F	F	F
0.4999700219999994	0.3080100119999969	0.1822699980000024	F	F	F
0.4422400199999998	0.5000000169999979	0.1126099949999997	F	F	F
0.3199488059650414	0.3750949223087237	0.2554588671121307	T	T	T
0.4422399970000015	0.3750000020000002	0.1096700009999978	F	F	F
0.3205361034682453	0.5002312166512805	0.2551082952916554	T	T	T
0.4999700190000027	0.4303799819999981	0.0370100009999987	F	F	F
0.3749800029999975	0.3077000190000021	0.1845699980000006	F	F	F
0.3076499940000019	0.3750599910000005	0.1096599999999981	F	F	F
0.4296336018619284	0.5000102281686362	0.2550171468998890	T	T	T
0.6248999909999995	0.3194800119999996	0.0372899979999985	F	F	F
0.7499199729999972	0.4420800179999986	0.1822600059999999	F	F	F
0.7499000509999973	0.3196600209999971	0.0370399999999975	F	F	F
0.6249100049999967	0.4423800049999969	0.1845799909999997	F	F	F
0.5576599750000000	0.5000800159999983	0.1126099949999997	F	F	F
0.6805044799264931	0.3753916652238134	0.2541969655718658	T	T	T
0.6249900039999972	0.4305599919999992	0.0373200009999977	F	F	F
0.7499899929999998	0.3079900230000021	0.1822600059999999	F	F	F
0.6922500240000034	0.5000000169999979	0.1126200049999966	F	F	F
0.5699333993074017	0.3755786118698686	0.2495423456970810	T	T	T

0.6922500270000000	0.3749800340000036	0.1096799930000003	F	F	F
0.5701743405538500	0.5005941801204022	0.2540992855232070	T	T	T
0.7499600310000005	0.4303500299999996	0.0370299989999978	F	F	F
0.6249900239999988	0.3076700029999984	0.1845699980000006	F	F	F
0.5576500530000033	0.3750800010000006	0.1096700009999978	F	F	F
0.6797639144165158	0.5005521532696589	0.2548936305428378	T	T	T
0.8748999780000020	0.3194899960000015	0.0372699999999995	F	F	F
0.9999099770000015	0.4420900020000005	0.1822200009999975	F	F	F
0.9999099540000032	0.3196899949999974	0.0369999999999990	F	F	F
0.8749000090000010	0.4423899889999987	0.1845499960000012	F	F	F
0.8076500040000028	0.5000699900000001	0.1126099949999997	F	F	F
0.9299289110861199	0.3751304090777061	0.2554127531427197	T	T	T
0.8749700830000009	0.4305400240000026	0.0372599989999998	F	F	F
0.9999799879999998	0.3079900230000021	0.1822299939999965	F	F	F
0.9422299519999981	0.5000000169999979	0.1125800000000012	F	F	F
0.8204885471059461	0.3750414470800594	0.2556193881894300	T	T	T
0.9422400130000028	0.3749700070000017	0.1096499989999984	F	F	F
0.8206784372498565	0.4999997281088925	0.2551930773904947	T	T	T
0.9999700520000019	0.4303199929999977	0.0370299989999978	F	F	F
0.8750000280000023	0.3076700029999984	0.1845499960000012	F	F	F
0.8076600070000026	0.3750700169999988	0.1096799930000003	F	F	F
0.9297028716402276	0.5001865069195138	0.2551422745275323	T	T	T
0.1249200099999968	0.5695000300000004	0.0372899979999985	F	F	F
0.2499099930000028	0.6920800469999975	0.1822900009999984	F	F	F
0.2499099950000030	0.5696600300000014	0.0370500009999972	F	F	F
0.1248900000000006	0.6923800349999993	0.1845799909999997	F	F	F
0.0576400030000030	0.7500699770000026	0.1126300060000034	F	F	F
0.1797150818586132	0.6250810803945663	0.2553636538746389	T	T	T
0.1249999979999998	0.6805800320000017	0.0373099999999980	F	F	F
0.2499800040000011	0.5579800049999974	0.1822800079999993	F	F	F
0.1922500069999984	0.7500000040000003	0.1126399979999988	F	F	F
0.0702212416441435	0.6249558632535784	0.2554038096995538	T	T	T
0.1922500000000014	0.6249700369999971	0.1096799930000003	F	F	F
0.0704147184198743	0.7499689881564595	0.2551598626522208	T	T	T
0.2499800020000009	0.6803500170000021	0.0370399999999975	F	F	F
0.1250000020000002	0.5576600070000026	0.1845699980000006	F	F	F
0.0576500000000024	0.6250700049999978	0.1096599999999981	F	F	F
0.1794256453152264	0.7500675271662223	0.2551695103479529	T	T	T
0.3749200159999972	0.5694699940000021	0.0373500000000035	F	F	F
0.4999299899999983	0.6920899890000030	0.1822900009999984	F	F	F
0.4999099739999977	0.5696600300000014	0.0370399999999975	F	F	F
0.3749000130000013	0.6923900609999976	0.1845900000000000	F	F	F
0.3076300070000002	0.7500800460000008	0.1126399979999988	F	F	F
0.4298286076221789	0.6251845970415724	0.2553415926199576	T	T	T

0.3749800029999975	0.6805600209999980	0.0373300019999974	F	F	F
0.4999799830000029	0.5579800049999974	0.1822800079999993	F	F	F
0.4422599899999966	0.7500000040000003	0.1126399979999988	F	F	F
0.3203525622189443	0.6249577795748809	0.2554113478105506	T	T	T
0.4422499840000000	0.6250000309999990	0.1097000039999969	F	F	F
0.3204038170778506	0.7499603869765412	0.2551864570554837	T	T	T
0.4999700059999981	0.6804100490000025	0.0370299989999978	F	F	F
0.3749899970000001	0.5576600070000026	0.1845799909999997	F	F	F
0.3076699849999969	0.6250600629999994	0.1097000039999969	F	F	F
0.4294166059818935	0.7501422819618221	0.2551850799696814	T	T	T
0.6249099950000030	0.5695000300000004	0.0373300019999974	F	F	F
0.7499399769999968	0.6920700209999993	0.1822499960000030	F	F	F
0.7498900200000023	0.5696500029999996	0.0370100009999987	F	F	F
0.6249200090000002	0.6924000030000030	0.1845900000000000	F	F	F
0.5576500370000019	0.7500800460000008	0.1126300060000034	F	F	F
0.6798810515598127	0.6252995465577278	0.2554438012575121	T	T	T
0.6249900070000010	0.6805600209999980	0.0372899979999985	F	F	F
0.7499899540000001	0.5579900310000028	0.1822600059999999	F	F	F
0.6922699949999966	0.7500000040000003	0.1126099949999997	F	F	F
0.5704421396427619	0.6251261245004198	0.2554907346414502	T	T	T
0.6922500049999982	0.6250000309999990	0.1096700009999978	F	F	F
0.5704644511347764	0.7501644925986733	0.2551621728823472	T	T	T
0.7499799850000031	0.6803800120000005	0.0370100009999987	F	F	F
0.6250000020000002	0.5576600070000026	0.1845799909999997	F	F	F
0.5576700230000000	0.6250800310000031	0.1096900029999972	F	F	F
0.6794759651548603	0.7502760233700146	0.2551659000109839	T	T	T
0.8749100160000012	0.5695000300000004	0.0372800009999992	F	F	F
0.9999099389999984	0.6920899890000030	0.1822800079999993	F	F	F
0.9999000159999980	0.5696900240000033	0.0370200019999984	F	F	F
0.8749300050000031	0.6923900609999976	0.1845900000000000	F	F	F
0.8076900590000022	0.7500600779999971	0.1125900019999975	F	F	F
0.9298236566423288	0.6251847949934793	0.2553961369664291	T	T	T
0.8749900110000013	0.6805400530000014	0.0372800009999992	F	F	F
0.9999900090000011	0.5579800049999974	0.1822399859999990	F	F	F
0.9422699400000027	0.7500100720000020	0.1126500000000021	F	F	F
0.8203612466823119	0.6250622362719390	0.2554882900579339	T	T	T
0.9422499750000028	0.6249900050000008	0.1096799930000003	F	F	F
0.8205280148072321	0.7501072529367813	0.2551330023556265	T	T	T
0.9999900059999973	0.6803500170000021	0.0370299989999978	F	F	F
0.8749900139999980	0.5576800170000027	0.1845699980000006	F	F	F
0.8076599680000029	0.6250700049999978	0.1096700009999978	F	F	F
0.9295354217140122	0.7502286692848860	0.2551700861402424	T	T	T
0.1249200090000002	0.8194700230000009	0.0373500000000035	F	F	F
0.2499199969999992	0.9421000449999966	0.1822900009999984	F	F	F

0.2498999900000030	0.8196800269999969	0.0370599979999966	F	F	F
0.1249100060000004	0.9424100589999966	0.1845600059999981	F	F	F
0.0576600010000021	0.0001000000000033	0.1125800000000012	F	F	F
0.1796277831777054	0.8750779318811936	0.2554119887579947	T	T	T
0.1250000029999967	0.9305700350000023	0.0372899979999985	F	F	F
0.2499800160000021	0.8079900179999981	0.1822900009999984	F	F	F
0.1922599970000007	0.0000199999999992	0.1126500000000021	F	F	F
0.0701634343706368	0.8749618675880444	0.2554023786040960	T	T	T
0.1922499869999967	0.8750000610000015	0.1097099970000031	F	F	F
0.0703469632590590	0.9999565849070391	0.2551062380862542	T	T	T
0.2499899979999967	0.9304100359999978	0.0370399999999975	F	F	F
0.1249900090000011	0.8076700200000033	0.1845699980000006	F	F	F
0.0576500000000024	0.8750900020000003	0.1096799930000003	F	F	F
0.1793535445682106	0.0000861264692152	0.2551697494967815	T	T	T
0.3748999940000033	0.8195000600000029	0.0373399989999967	F	F	F
0.4999200099999968	0.9420899759999983	0.1822999930000009	F	F	F
0.4998999769999983	0.8196400060000002	0.0370599979999966	F	F	F
0.3748999999999967	0.9424200429999985	0.1845900000000000	F	F	F
0.3076599979999983	0.0000900000000001	0.1126300060000034	F	F	F
0.4296028074985394	0.8750778307549102	0.2554192694082273	T	T	T
0.3749899820000024	0.9305999870000008	0.0373500000000035	F	F	F
0.4999899780000021	0.8079900179999981	0.1822900009999984	F	F	F
0.4422599970000007	0.0000199999999992	0.1126600010000018	F	F	F
0.3201127206343844	0.8750004313389784	0.2553744891867205	T	T	T
0.4422400289999970	0.8749800080000014	0.1097199980000028	F	F	F
0.3203850282479148	0.0000110296459590	0.2551990679740296	T	T	T
0.4999799929999966	0.9303600309999993	0.0370599979999966	F	F	F
0.3749999930000030	0.8076700200000033	0.1845799909999997	F	F	F
0.3076600010000021	0.8750800179999985	0.1097099970000031	F	F	F
0.4293723138435545	0.0000891850465971	0.2552329486396593	T	T	T
0.6249000059999972	0.8194900760000010	0.0373000029999986	F	F	F
0.7499199800000014	0.9420800760000034	0.1822800079999993	F	F	F
0.7499100240000018	0.8196400060000002	0.0370200019999984	F	F	F
0.6249100289999987	0.9423999899999984	0.1845699980000006	F	F	F
0.5576400100000001	0.0000900000000001	0.1126300060000034	F	F	F
0.6797626268042704	0.8752400973694018	0.2553528488021456	T	T	T
0.6249799940000003	0.9305700350000023	0.0373600019999998	F	F	F
0.7499999830000021	0.8079900179999981	0.1822499960000030	F	F	F
0.6922600520000017	0.0000100000000032	0.1126399979999988	F	F	F
0.5702279937966527	0.8751743387774203	0.2553569762379427	T	T	T
0.6922500249999999	0.8749700239999996	0.1096700009999978	F	F	F
0.5704306979009504	0.0001055654803441	0.2551870923836849	T	T	T
0.7499700219999994	0.9303500469999975	0.0370399999999975	F	F	F
0.6250099969999994	0.8076700200000033	0.1845499960000012	F	F	F

0.5576700180000032	0.8750700339999966	0.1097000039999969	F	F	F
0.6794724026243047	0.0002702684242210	0.2551936518089518	T	T	T
0.8749099599999965	0.8194800070000028	0.0373000029999986	F	F	F
0.9999200700000017	0.9421100289999984	0.1822299939999965	F	F	F
0.9999200379999991	0.8196999950000006	0.0370399999999975	F	F	F
0.8749000250000023	0.9423999899999984	0.1845400039999987	F	F	F
0.8076400309999983	0.0000900000000001	0.1126300060000034	F	F	F
0.9297364723399012	0.8752426294693206	0.2553640896005121	T	T	T
0.8749700899999979	0.9305400829999968	0.0372899979999985	F	F	F
0.9999899959999965	0.8080099869999984	0.1822800079999993	F	F	F
0.9422299539999983	0.0000100000000032	0.1125900019999975	F	F	F
0.8202597645773266	0.8750924057092274	0.2553489919265632	T	T	T
0.9422400460000020	0.8750000610000015	0.1096700009999978	F	F	F
0.8203758039882639	0.0000570350817882	0.2551684260441097	T	T	T
0.9999700090000019	0.9303600309999993	0.0370399999999975	F	F	F
0.8750100270000019	0.8076800039999981	0.1845600059999981	F	F	F
0.8076600139999996	0.8750700339999966	0.1096799930000003	F	F	F
0.9294207402785811	0.0002156280406435	0.2551291447098771	T	T	T
0.4822059297620371	0.3200595078140589	0.3497819277322471	T	T	T
0.4336420412098400	0.4039787921461159	0.3727904107899153	T	T	T
0.5186134925452437	0.4707029591079561	0.3692834534987389	T	T	T
0.6073729933853419	0.4251737500910212	0.3367641819060673	T	T	T
0.6215702726394033	0.3222179503004787	0.3290349602563634	T	T	T
0.5238187249567376	0.3649317344949228	0.3513371417744289	T	T	T
0.4926761121413859	0.4170197089197418	0.3655138646189263	T	T	T
0.5839697638961545	0.3702586939987069	0.3412239163397125	T	T	T
0.4292146756718245	0.3471265137893236	0.3615680276615861	T	T	T
0.5738564754943155	0.4718691126075933	0.3526920099564448	T	T	T
0.4846756468461822	0.2829600264696208	0.3278526303772029	T	T	T
0.3876959414848067	0.3223774733974897	0.3612166239901079	T	T	T
0.5959211938014630	0.5149533140464362	0.3504498429289920	T	T	T
0.6649761045751683	0.3313583872589665	0.3376918557689444	T	T	T
0.6081695741145781	0.2824484985290328	0.3436065793619432	T	T	T

Data S2. CONTCAR of adenine adsorbed on C-100.

CONTCAR-adenine adsorbed on Co_3O_4-(111)

1.00000000000000
22.5426006317000009 0.0000000000000000 0.0000000000000000
-11.2648970034999998 19.5256979472999994 0.0000000000000000
0.0000000000000000 0.0000000000000000 28.6224002838000011
Co O N C H
192 256 5 5 5
Selective dynamics
Direct

0.1668999919999976	0.0830999960000014	0.0232800010000034	F	F	F
0.0834599969999985	0.1665399930000007	0.1798300000000026	F	F	F
0.0415299969999978	0.0824600010000012	0.0799200020000015	F	F	F
0.2081000040000021	0.1659599939999978	0.2431900139999996	F	F	F
0.1675299999999993	0.2084700030000022	0.0799299979999972	F	F	F
0.0840400040000020	0.0418899989999986	0.2431900139999996	F	F	F
0.1668300030000012	0.0831499980000032	0.1420000099999967	F	F	F
0.0833696508700193	0.1665472409696183	0.2941504502794575	T	T	T
0.0414899940000026	0.2084999920000001	0.0799200020000015	F	F	F
0.2081000089999989	0.0418999999999983	0.2432200009999974	F	F	F
0.0834799969999978	0.1665299920000010	0.0030100000000033	F	F	F
0.0000899989999965	0.2498999929999997	0.1612100050000009	F	F	F
0.4168999819999968	0.0830999960000014	0.0232800010000034	F	F	F
0.3334600020000025	0.1665399930000007	0.1798300000000026	F	F	F
0.2915300020000018	0.0824600010000012	0.0799200020000015	F	F	F
0.4580999940000012	0.1659599939999978	0.2431900139999996	F	F	F
0.4175299999999993	0.2084700030000022	0.0799299979999972	F	F	F
0.3340399770000033	0.0418899989999986	0.2431900139999996	F	F	F
0.4168299930000003	0.0831499980000032	0.1420000099999967	F	F	F
0.3333303706891463	0.1664181134228004	0.2941072719431009	T	T	T
0.2914899840000018	0.2084999920000001	0.0799200020000015	F	F	F
0.4580999880000007	0.0418999999999983	0.2432200009999974	F	F	F
0.3334800080000022	0.1665299920000010	0.0030100000000033	F	F	F
0.2500899989999965	0.2498999929999997	0.1612100050000009	F	F	F
0.6668999600000021	0.0830999960000014	0.0232800010000034	F	F	F
0.5834599809999972	0.1665399930000007	0.1798300000000026	F	F	F
0.5415300020000018	0.0824600010000012	0.0799200020000015	F	F	F
0.7081000149999994	0.1659599939999978	0.2431900139999996	F	F	F
0.6675299790000011	0.2084700030000022	0.0799299979999972	F	F	F
0.5840399770000033	0.0418899989999986	0.2431900139999996	F	F	F
0.6668300139999985	0.0831499980000032	0.1420000099999967	F	F	F
0.5834018870772796	0.1664557930911784	0.2941547853226460	T	T	T
0.5414900259999982	0.2084999920000001	0.0799200020000015	F	F	F
0.7081000089999989	0.0418999999999983	0.2432200009999974	F	F	F

0.5834799860000004	0.1665299920000010	0.0030100000000033	F	F	F
0.5000899779999983	0.2498999929999997	0.1612100050000009	F	F	F
0.9168999389999968	0.0830999960000014	0.0232800010000034	F	F	F
0.8334599180000026	0.1665399930000007	0.1798300000000026	F	F	F
0.7915300230000000	0.0824600010000012	0.0799200020000015	F	F	F
0.9581000359999976	0.1659599939999978	0.2431900139999996	F	F	F
0.9175299999999993	0.2084700030000022	0.0799299979999972	F	F	F
0.8340399980000015	0.0418899989999986	0.2431900139999996	F	F	F
0.9168299930000003	0.0831499980000032	0.1420000099999967	F	F	F
0.8334177169701429	0.1665597896680379	0.2941930849081382	T	T	T
0.7914900050000000	0.2084999920000001	0.0799200020000015	F	F	F
0.9580999880000007	0.0418999999999983	0.2432200009999974	F	F	F
0.8334799650000022	0.1665299920000010	0.0030100000000033	F	F	F
0.7500899989999965	0.2498999929999997	0.1612100050000009	F	F	F
0.1668999839999969	0.3330999869999971	0.0232800010000034	F	F	F
0.0834599989999987	0.4165300259999967	0.1798300000000026	F	F	F
0.0415299909999973	0.3324599849999998	0.0799200020000015	F	F	F
0.2080999989999981	0.4159599910000011	0.2431900139999996	F	F	F
0.1675299830000014	0.4584699749999999	0.0799299979999972	F	F	F
0.0840399960000013	0.2918899809999971	0.2431900139999996	F	F	F
0.1668300010000010	0.3331500009999999	0.1420000099999967	F	F	F
0.0834694359209014	0.4166074088023297	0.2942096328025497	T	T	T
0.0414900009999997	0.4585000129999983	0.0799200020000015	F	F	F
0.2080999909999974	0.2918999939999978	0.2432200009999974	F	F	F
0.0834800100000024	0.4165300259999967	0.0030100000000033	F	F	F
0.0000899950000033	0.4999000139999978	0.1612100050000009	F	F	F
0.4168999839999969	0.3330999869999971	0.0232800010000034	F	F	F
0.3334599860000012	0.4165399899999969	0.1798300000000026	F	F	F
0.2915300009999982	0.3324599849999998	0.0799200020000015	F	F	F
0.4580999779999999	0.4159599910000011	0.2431900139999996	F	F	F
0.4175299930000023	0.4584699749999999	0.0799299979999972	F	F	F
0.3340399750000032	0.2918899809999971	0.2431900139999996	F	F	F
0.4168299800000028	0.3331500009999999	0.1420000099999967	F	F	F
0.3337672639738108	0.4175299081604700	0.2945274311080686	T	T	T
0.2914899800000015	0.4585000129999983	0.0799200020000015	F	F	F
0.4580999699999992	0.2918999939999978	0.2432200009999974	F	F	F
0.3334800100000024	0.4165300259999967	0.0030100000000033	F	F	F
0.2500899950000033	0.4999000139999978	0.1612100050000009	F	F	F
0.6668999629999988	0.3330999869999971	0.0232800010000034	F	F	F
0.5834599439999977	0.4165399899999969	0.1798300000000026	F	F	F
0.5415300009999982	0.3324599849999998	0.0799200020000015	F	F	F
0.7080999779999999	0.4159599910000011	0.2431900139999996	F	F	F
0.6675299719999970	0.4584699749999999	0.0799299979999972	F	F	F
0.5840399750000032	0.2918899809999971	0.2431900139999996	F	F	F

0.6668300219999992	0.3331500009999999	0.1420000099999967	F	F	F
0.5837432984174691	0.4163350310471298	0.2931862478669993	T	T	T
0.5414900219999979	0.4585000129999983	0.0799200020000015	F	F	F
0.7081000129999993	0.2918999939999978	0.2432200009999974	F	F	F
0.5834800100000024	0.4165300259999967	0.0030100000000033	F	F	F
0.5000899739999980	0.4999000139999978	0.1612100050000009	F	F	F
0.9168999839999969	0.3330999869999971	0.0232800010000034	F	F	F
0.8334599650000030	0.4165399899999969	0.1798300000000026	F	F	F
0.7915300220000034	0.3324599849999998	0.0799200020000015	F	F	F
0.9580999989999981	0.4159599910000011	0.2431900139999996	F	F	F
0.9175299509999988	0.4584699749999999	0.0799299979999972	F	F	F
0.8340399539999979	0.2918899809999971	0.2431900139999996	F	F	F
0.9168299589999975	0.3331500009999999	0.1420000099999967	F	F	F
0.8335275277638295	0.4166010242675982	0.2941315452161178	T	T	T
0.7914900219999979	0.4585000129999983	0.0799200020000015	F	F	F
0.9581000339999974	0.2918999939999978	0.2432200009999974	F	F	F
0.8334800310000006	0.4165300259999967	0.0030100000000033	F	F	F
0.7500899739999980	0.4999000139999978	0.1612100050000009	F	F	F
0.1668999910000011	0.5830900200000002	0.0232800010000034	F	F	F
0.0834599829999974	0.6665400110000022	0.1798300000000026	F	F	F
0.0415299940000011	0.5824599580000012	0.0799200020000015	F	F	F
0.2080999949999978	0.6659600119999993	0.2431900139999996	F	F	F
0.1675299890000019	0.7084699970000017	0.0799299979999972	F	F	F
0.0840400049999985	0.5418900270000009	0.2431900139999996	F	F	F
0.1668299849999997	0.5831499980000032	0.1420000099999967	F	F	F
0.0834335345075326	0.6665605064792232	0.2941892919096336	T	T	T
0.0414899760000011	0.7085000350000001	0.0799200020000015	F	F	F
0.2080999969999979	0.5418999910000011	0.2432200009999974	F	F	F
0.0834799820000001	0.6665299989999980	0.0030100000000033	F	F	F
0.0000899879999992	0.7498999869999992	0.1612100050000009	F	F	F
0.4168999919999976	0.5831000319999973	0.0232800010000034	F	F	F
0.3334599829999974	0.6665400110000022	0.1798300000000026	F	F	F
0.2915299940000011	0.5824599580000012	0.0799200020000015	F	F	F
0.4580999739999996	0.6659600119999993	0.2431900139999996	F	F	F
0.4175300109999966	0.7084699970000017	0.0799299979999972	F	F	F
0.3340399829999967	0.5418900270000009	0.2431900139999996	F	F	F
0.4168299640000015	0.5831499980000032	0.1420000099999967	F	F	F
0.3335676265920104	0.6664799593408446	0.2944899369371503	T	T	T
0.2914899760000011	0.7085000350000001	0.0799200020000015	F	F	F
0.4580999759999997	0.5418999910000011	0.2432200009999974	F	F	F
0.3334799820000001	0.6665299989999980	0.0030100000000033	F	F	F
0.2500900100000010	0.7498999869999992	0.1612100050000009	F	F	F
0.6668999500000012	0.5831000319999973	0.0232800010000034	F	F	F
0.5834599619999992	0.6665400110000022	0.1798300000000026	F	F	F

0.5415299940000011	0.5824599580000012	0.0799200020000015	F	F	F
0.7080999949999978	0.6659600119999993	0.2431900139999996	F	F	F
0.6675299890000019	0.7084699970000017	0.0799299979999972	F	F	F
0.5840399829999967	0.5418900270000009	0.2431900139999996	F	F	F
0.6668299849999997	0.5831499980000032	0.1420000099999967	F	F	F
0.5830532195893496	0.6663637540398570	0.2957059811045403	T	T	T
0.5414900190000012	0.7085000350000001	0.0799200020000015	F	F	F
0.7080999759999997	0.5418999910000011	0.2432200009999974	F	F	F
0.5834799610000019	0.6665299989999980	0.0030100000000033	F	F	F
0.5000899879999992	0.7498999869999992	0.1612100050000009	F	F	F
0.9168999709999994	0.5831000319999973	0.0232800010000034	F	F	F
0.8334599399999973	0.6665400110000022	0.1798300000000026	F	F	F
0.7915300160000029	0.5824599580000012	0.0799200020000015	F	F	F
0.9581000160000031	0.6659600119999993	0.2431900139999996	F	F	F
0.9175299679999966	0.7084699970000017	0.0799299979999972	F	F	F
0.8340399410000003	0.5418900270000009	0.2431900139999996	F	F	F
0.9168299640000015	0.5831499980000032	0.1420000099999967	F	F	F
0.8334939932005395	0.6667885733981977	0.2941978655895843	T	T	T
0.7914899980000030	0.7085000350000001	0.0799200020000015	F	F	F
0.9580999969999979	0.5418999910000011	0.2432200009999974	F	F	F
0.8334799820000001	0.6665299989999980	0.0030100000000033	F	F	F
0.7500900100000010	0.7498999869999992	0.1612100050000009	F	F	F
0.1668999429999971	0.8331000049999986	0.0232800010000034	F	F	F
0.0834600210000005	0.9165400329999969	0.1798300000000026	F	F	F
0.0415300120000026	0.8324599789999994	0.0799200020000015	F	F	F
0.2080999880000007	0.9159599850000006	0.2431900139999996	F	F	F
0.1675300070000034	0.9584700179999999	0.0799299979999972	F	F	F
0.0840399759999997	0.7918899989999986	0.2431900139999996	F	F	F
0.1668300030000012	0.8331500190000014	0.1420000099999967	F	F	F
0.0834108529566145	0.9165588036243041	0.2941882739036045	T	T	T
0.0414899700000007	0.9585000069999978	0.0799200020000015	F	F	F
0.2080999690000027	0.7918999629999988	0.2432200009999974	F	F	F
0.0834799539999977	0.9165299710000028	0.0030100000000033	F	F	F
0.0000899389999987	0.9998999589999968	0.1612100050000009	F	F	F
0.4168999640000024	0.8331000049999986	0.0232800010000034	F	F	F
0.3334599639999993	0.9165299710000028	0.1798300000000026	F	F	F
0.2915300120000026	0.8324599789999994	0.0799200020000015	F	F	F
0.4580999670000026	0.9159599850000006	0.2431900139999996	F	F	F
0.4175300280000016	0.9584700179999999	0.0799299979999972	F	F	F
0.3340399550000015	0.7918899989999986	0.2431900139999996	F	F	F
0.4168299809999993	0.8331500190000014	0.1420000099999967	F	F	F
0.3334197294026949	0.9165447442715708	0.2941894582342134	T	T	T
0.2914899700000007	0.9585000069999978	0.0799200020000015	F	F	F
0.4580999470000009	0.7918999629999988	0.2432200009999974	F	F	F

0.3334799750000030	0.9165299710000028	0.0030100000000033	F	F	F
0.2500899599999968	0.9998999589999968	0.1612100050000009	F	F	F
0.6668999429999971	0.8331000049999986	0.0232800010000034	F	F	F
0.5834599789999970	0.9165400329999969	0.1798300000000026	F	F	F
0.5415300120000026	0.8324599789999994	0.0799200020000015	F	F	F
0.7080999880000007	0.9159599850000006	0.2431900139999996	F	F	F
0.6675300070000034	0.9584700179999999	0.0799299979999972	F	F	F
0.5840399550000015	0.7918899989999986	0.2431900139999996	F	F	F
0.6668300030000012	0.8331500190000014	0.1420000099999967	F	F	F
0.5833632597331121	0.9165141554586630	0.2942256527894864	T	T	T
0.5414900119999970	0.9585000069999978	0.0799200020000015	F	F	F
0.7080999690000027	0.7918999629999988	0.2432200009999974	F	F	F
0.5834799539999977	0.9165299710000028	0.0030100000000033	F	F	F
0.5000899389999987	0.9998999589999968	0.1612100050000009	F	F	F
0.9168999219999989	0.8331000049999986	0.0232800010000034	F	F	F
0.8334599579999988	0.9165400329999969	0.1798300000000026	F	F	F
0.7915300330000008	0.8324599789999994	0.0799200020000015	F	F	F
0.9581000089999989	0.9159599850000006	0.2431900139999996	F	F	F
0.9175299859999981	0.9584700179999999	0.0799299979999972	F	F	F
0.8340399340000033	0.7918899989999986	0.2431900139999996	F	F	F
0.9168299809999993	0.8331500190000014	0.1420000099999967	F	F	F
0.8334221787901077	0.9165777555909050	0.2941970593119222	T	T	T
0.7914899909999988	0.9585000069999978	0.0799200020000015	F	F	F
0.9580999900000009	0.7918999629999988	0.2432200009999974	F	F	F
0.8334799750000030	0.9165299710000028	0.0030100000000033	F	F	F
0.7500899599999968	0.9998999589999968	0.1612100050000009	F	F	F
0.2114599989999988	0.0385299989999979	0.1190500060000019	F	F	F
0.1265971371650535	0.1233985871824941	0.2783836125006684	T	T	T
0.0047899990000033	0.1271499989999967	0.0387400030000009	F	F	F
0.1728399980000006	0.2113299960000035	0.2022899959999975	F	F	F
0.2115099939999965	0.1724699990000005	0.1191200009999989	F	F	F
0.1266029191949216	0.0029406523348980	0.2784155534768602	T	T	T
0.0775099949999998	0.0384799970000032	0.1191200009999989	F	F	F
0.2469443806991547	0.1233750560828641	0.2784101860454815	T	T	T
0.0001699979999970	0.2498199899999989	0.0462899999999991	F	F	F
0.1667300009999977	0.0832699959999985	0.2062699860000023	F	F	F
0.1228499999999997	0.2452100120000011	0.0387400030000009	F	F	F
0.0386699969999995	0.0771600019999994	0.2022899959999975	F	F	F
0.1229199989999969	0.1270699960000030	0.0387100040000021	F	F	F
0.0387299850000034	0.2112699930000019	0.2023200000000003	F	F	F
0.0835199970000033	0.1664700019999970	0.1144300050000027	F	F	F
0.2500902306712283	0.9999170213837245	0.2753388542363992	T	T	T
0.4614600199999970	0.0385299989999979	0.1190500060000019	F	F	F
0.3766853415084219	0.1234007363323066	0.2783851527384245	T	T	T

0.2547900140000010	0.1271499989999967	0.0387400030000009	F	F	F
0.4228399879999998	0.2113299960000035	0.2022899959999975	F	F	F
0.4615100150000018	0.1724699990000005	0.1191200009999989	F	F	F
0.3766007701931426	0.0029313788233104	0.2784100165486083	T	T	T
0.3275099949999998	0.0384799970000032	0.1191200009999989	F	F	F
0.4970041926001418	0.1233851408047526	0.2784124297832565	T	T	T
0.2501599929999969	0.2498199899999989	0.0462899999999991	F	F	F
0.4167299590000013	0.0832699959999985	0.2062699860000023	F	F	F
0.3728499999999997	0.2452100120000011	0.0387400030000009	F	F	F
0.2886700020000035	0.0771600019999994	0.2022899959999975	F	F	F
0.3729200040000009	0.1270699960000030	0.0387100040000021	F	F	F
0.2887299790000029	0.2112699930000019	0.2023200000000003	F	F	F
0.3335199819999985	0.1664700019999970	0.1144300050000027	F	F	F
0.5000904907429379	0.9999156022880057	0.2753314438847947	T	T	T
0.7114599989999988	0.0385299989999979	0.1190500060000019	F	F	F
0.6266442254965164	0.1233543847059760	0.2783962293452009	T	T	T
0.5047899719999975	0.1271499989999967	0.0387400030000009	F	F	F
0.6728400089999980	0.2113299960000035	0.2022899959999975	F	F	F
0.7115099939999965	0.1724699990000005	0.1191200009999989	F	F	F
0.6266070123307603	0.0029149710395611	0.2784069565267375	T	T	T
0.5775099949999998	0.0384799970000032	0.1191200009999989	F	F	F
0.7470255028273988	0.1233722204539682	0.2784104814143575	T	T	T
0.5001699769999988	0.2498199899999989	0.0462899999999991	F	F	F
0.6667299799999995	0.0832699959999985	0.2062699860000023	F	F	F
0.6228500209999979	0.2452100120000011	0.0387400030000009	F	F	F
0.5386699809999982	0.0771600019999994	0.2022899959999975	F	F	F
0.6229199619999974	0.1270699960000030	0.0387100040000021	F	F	F
0.5387300009999976	0.2112699930000019	0.2023200000000003	F	F	F
0.5835200240000020	0.1664700019999970	0.1144300050000027	F	F	F
0.7500899872125580	0.9999076752285357	0.2753294915405873	T	T	T
0.9614600199999970	0.0385299989999979	0.1190500060000019	F	F	F
0.8766031737756890	0.1233948958455894	0.2783866881057920	T	T	T
0.7547899509999993	0.1271499989999967	0.0387400030000009	F	F	F
0.9228399449999998	0.2113299960000035	0.2022899959999975	F	F	F
0.9615099729999983	0.1724699990000005	0.1191200009999989	F	F	F
0.8766137548732734	0.0029681384099735	0.2784139089281436	T	T	T
0.8275100170000016	0.0384799970000032	0.1191200009999989	F	F	F
0.9969896292967327	0.1233614651334572	0.2784072426776283	T	T	T
0.7501699560000006	0.2498199899999989	0.0462899999999991	F	F	F
0.9167299590000013	0.0832699959999985	0.2062699860000023	F	F	F
0.8728499999999997	0.2452100120000011	0.0387400030000009	F	F	F
0.7886700020000035	0.0771600019999994	0.2022899959999975	F	F	F
0.8729199830000027	0.1270699960000030	0.0387100040000021	F	F	F
0.7887299790000029	0.2112699930000019	0.2023200000000003	F	F	F

0.8335200450000002	0.1664700019999970	0.1144300050000027	F	F	F
0.0000896077349570	0.9999132790028131	0.2753313082401193	T	T	T
0.2114599820000009	0.2885299929999974	0.1190500060000019	F	F	F
0.1266072797177020	0.3733767115145172	0.2783848348490240	T	T	T
0.0047899710000010	0.3771499839999990	0.0387400030000009	F	F	F
0.1728399810000028	0.4613299680000011	0.2022899959999975	F	F	F
0.2115099780000023	0.4224699959999967	0.1191200009999989	F	F	F
0.1266478314505112	0.2529734651981086	0.2784252414778143	T	T	T
0.0775099889999993	0.2884800029999965	0.1191200009999989	F	F	F
0.2473315222602656	0.3733387821471504	0.2782102487076101	T	T	T
0.0001699950000003	0.4998200109999971	0.0462899999999991	F	F	F
0.1667299909999969	0.3332700059999993	0.2062699860000023	F	F	F
0.1228499830000018	0.4951999970000003	0.0387400030000009	F	F	F
0.0386699829999984	0.3271599919999986	0.2022899959999975	F	F	F
0.1229199949999966	0.3770700050000002	0.0387100040000021	F	F	F
0.0387299850000034	0.4612699899999981	0.2023200000000003	F	F	F
0.0835200080000007	0.4164699990000003	0.1144300050000027	F	F	F
0.2501143843369770	0.2499341694388151	0.2754185004682654	T	T	T
0.4614600029999991	0.2885299929999974	0.1190500060000019	F	F	F
0.3762847705790516	0.3731333472943529	0.2785919244189085	T	T	T
0.2547899919999992	0.3771499839999990	0.0387400030000009	F	F	F
0.4228399599999975	0.4613299680000011	0.2022899959999975	F	F	F
0.4615099990000004	0.4224699959999967	0.1191200009999989	F	F	F
0.3766496197457721	0.2529773549570826	0.2785363057583991	T	T	T
0.3275099840000024	0.2884800029999965	0.1191200009999989	F	F	F
0.4966328994796302	0.3735506552961362	0.2790253098594205	T	T	T
0.2501599900000002	0.4998200109999971	0.0462899999999991	F	F	F
0.4167299699999987	0.3332700059999993	0.2062699860000023	F	F	F
0.3728499730000010	0.4951999970000003	0.0387400030000009	F	F	F
0.2886699829999984	0.3271599919999986	0.2022899959999975	F	F	F
0.3729199949999966	0.3770700050000002	0.0387100040000021	F	F	F
0.2887299850000034	0.4612699899999981	0.2023200000000003	F	F	F
0.3335199870000025	0.4164699990000003	0.1144300050000027	F	F	F
0.5000787772364890	0.2499792866200912	0.2754107224816676	T	T	T
0.7114599610000027	0.2885299929999974	0.1190500060000019	F	F	F
0.6269163355082087	0.3730136170969131	0.2784287512188814	T	T	T
0.5047899710000010	0.3771499839999990	0.0387400030000009	F	F	F
0.6728399599999975	0.4613299680000011	0.2022899959999975	F	F	F
0.7115099990000004	0.4224699959999967	0.1191200009999989	F	F	F
0.6265820806378287	0.2528491367730319	0.2784013331193054	T	T	T
0.5775100050000006	0.2884800029999965	0.1191200009999989	F	F	F
0.7471336610400360	0.3733975544783370	0.2784064148635538	T	T	T
0.5001699740000021	0.4998200109999971	0.0462899999999991	F	F	F
0.6667299909999969	0.3332700059999993	0.2062699860000023	F	F	F

0.6228500260000018	0.4952100089999973	0.0387400030000009	F	F	F
0.5386699829999984	0.3271599919999986	0.2022899959999975	F	F	F
0.6229199730000019	0.3770700050000002	0.0387100040000021	F	F	F
0.5387300060000015	0.4612699899999981	0.2023200000000003	F	F	F
0.5835199870000025	0.4164699990000003	0.1144300050000027	F	F	F
0.7500906599717507	0.2499111483991477	0.2753327814582595	T	T	T
0.9614599820000009	0.2885299929999974	0.1190500060000019	F	F	F
0.8766331212016186	0.3733542229097551	0.2783894979179379	T	T	T
0.7547899500000028	0.3771499839999990	0.0387400030000009	F	F	F
0.9228399810000028	0.4613299680000011	0.2022899959999975	F	F	F
0.9615100210000023	0.4224699959999967	0.1191200009999989	F	F	F
0.8766150622503058	0.2529444879286528	0.2784065463850055	T	T	T
0.8275100259999988	0.2884800029999965	0.1191200009999989	F	F	F
0.9970733787948169	0.3733941085919946	0.2784005528753648	T	T	T
0.7501699740000021	0.4998200109999971	0.0462899999999991	F	F	F
0.9167299280000023	0.3332700059999993	0.2062699860000023	F	F	F
0.8728500470000000	0.4952100089999973	0.0387400030000009	F	F	F
0.7886700039999965	0.3271599919999986	0.2022899959999975	F	F	F
0.8729199949999966	0.3770700050000002	0.0387100040000021	F	F	F
0.7887299850000034	0.4612699899999981	0.2023200000000003	F	F	F
0.8335200080000007	0.4164699990000003	0.1144300050000027	F	F	F
0.0000919068479768	0.2499131944104117	0.2753310938277351	T	T	T
0.2114599869999978	0.5385299900000007	0.1190500060000019	F	F	F
0.1266229574932052	0.6233961395774655	0.2783813631808348	T	T	T
0.0047899890000025	0.6271500049999972	0.0387400030000009	F	F	F
0.1728399770000024	0.7113299889999993	0.2022899959999975	F	F	F
0.2115099920000034	0.6724699680000015	0.1191200009999989	F	F	F
0.1266209920068767	0.5029921005317607	0.2784012257028602	T	T	T
0.0775099769999983	0.5384799750000013	0.1191200009999989	F	F	F
0.2471658116935872	0.6234863692668675	0.2784227704196907	T	T	T
0.0001699450000032	0.7498199839999984	0.0462899999999991	F	F	F
0.1667299750000026	0.5832700030000026	0.2062699860000023	F	F	F
0.1228499770000013	0.7452099819999987	0.0387400030000009	F	F	F
0.0386700009999998	0.5771600140000004	0.2022899959999975	F	F	F
0.1229199790000024	0.6270700019999964	0.0387100040000021	F	F	F
0.0387299779999992	0.7112699629999994	0.2023200000000003	F	F	F
0.0835199799999984	0.6664699720000016	0.1144300050000027	F	F	F
0.2500733904758759	0.4998728980619439	0.2752787551976364	T	T	T
0.4614600080000031	0.5385299900000007	0.1190500060000019	F	F	F
0.3766350670692886	0.6234248887698683	0.2782590026515293	T	T	T
0.2547900100000007	0.6271500049999972	0.0387400030000009	F	F	F
0.4228399559999971	0.7113299889999993	0.2022899959999975	F	F	F
0.4615100139999981	0.6724699680000015	0.1191200009999989	F	F	F
0.3768898709509177	0.5036404761068110	0.2778323268885187	T	T	T

0.3275099769999983	0.5384799750000013	0.1191200009999989	F	F	F
0.4969387089949282	0.6232825209054056	0.2778387132683269	T	T	T
0.2501599610000014	0.7498199839999984	0.0462899999999991	F	F	F
0.4167299539999973	0.5832700030000026	0.2062699860000023	F	F	F
0.3728499560000031	0.7452099819999987	0.0387400030000009	F	F	F
0.2886700009999998	0.5771600140000004	0.2022899959999975	F	F	F
0.3729199790000024	0.6270700019999964	0.0387100040000021	F	F	F
0.2887299779999992	0.7112699629999994	0.2023200000000003	F	F	F
0.3335199590000002	0.6664699720000016	0.1144300050000027	F	F	F
0.5001362867284360	0.4998575903760296	0.2753993540819442	T	T	T
0.7114600080000031	0.5385299900000007	0.1190500060000019	F	F	F
0.6263953265387863	0.6235788934613182	0.2776057654604440	T	T	T
0.5047899890000025	0.6271500049999972	0.0387400030000009	F	F	F
0.6728399770000024	0.7113299889999993	0.2022899959999975	F	F	F
0.7115099920000034	0.6724699680000015	0.1191200009999989	F	F	F
0.6265862679935147	0.5029980084859464	0.2779852166223818	T	T	T
0.5775099979999965	0.5384799750000013	0.1191200009999989	F	F	F
0.7469558429792083	0.6232047538681923	0.2786074232153025	T	T	T
0.5001699450000032	0.7498199839999984	0.0462899999999991	F	F	F
0.6667299539999973	0.5832700030000026	0.2062699860000023	F	F	F
0.6228499770000013	0.7452099819999987	0.0387400030000009	F	F	F
0.5386700009999998	0.5771600140000004	0.2022899959999975	F	F	F
0.6229199570000006	0.6270700019999964	0.0387100040000021	F	F	F
0.5387299989999974	0.7112699629999994	0.2023200000000003	F	F	F
0.5835199799999984	0.6664699720000016	0.1144300050000027	F	F	F
0.7499914817813838	0.4999165392143237	0.2755145989568361	T	T	T
0.9614600290000013	0.5385299900000007	0.1190500060000019	F	F	F
0.8764780547556791	0.6233953818126853	0.2783991397157460	T	T	T
0.7547899679999972	0.6271500049999972	0.0387400030000009	F	F	F
0.9228399559999971	0.7113299889999993	0.2022899959999975	F	F	F
0.9615100139999981	0.6724699680000015	0.1191200009999989	F	F	F
0.8766154962112899	0.5030031896035168	0.2784147468798520	T	T	T
0.8275100190000018	0.5384799750000013	0.1191200009999989	F	F	F
0.9970444688672917	0.6234042218161057	0.2784107655813011	T	T	T
0.7501699239999979	0.7498199839999984	0.0462899999999991	F	F	F
0.9167299750000026	0.5832700030000026	0.2062699860000023	F	F	F
0.8728499979999995	0.7452099819999987	0.0387400030000009	F	F	F
0.7886700219999980	0.5771600140000004	0.2022899959999975	F	F	F
0.8729200000000006	0.6270700019999964	0.0387100040000021	F	F	F
0.7887299779999992	0.7112699629999994	0.2023200000000003	F	F	F
0.8335200009999966	0.6664699720000016	0.1144300050000027	F	F	F
0.0000804424254652	0.4999115704580033	0.2753296956685329	T	T	T
0.2114599590000026	0.7885299619999984	0.1190500060000019	F	F	F
0.1266025856856094	0.8734046639515570	0.2783804654329939	T	T	T

0.0047900060000003	0.8771500269999990	0.0387400030000009	F	F	F
0.1728399940000003	0.9613300110000012	0.2022899959999975	F	F	F
0.2115099860000029	0.9224699410000028	0.1191200009999989	F	F	F
0.1265983512581740	0.7529380688842556	0.2784140222511021	T	T	T
0.0775099829999988	0.7884699840000025	0.1191200009999989	F	F	F
0.2470329992021240	0.8733915416570042	0.2784046995018225	T	T	T
0.0001699390000027	0.9998199560000032	0.0462899999999991	F	F	F
0.1667299470000003	0.8332699750000003	0.2062699860000023	F	F	F
0.1228499699999972	0.9952099540000034	0.0387400030000009	F	F	F
0.0386700179999977	0.8271600349999986	0.2022899959999975	F	F	F
0.1229199750000021	0.8770700239999982	0.0387100040000021	F	F	F
0.0387299499999969	0.9612699349999971	0.2023200000000003	F	F	F
0.0835199970000033	0.9164699929999998	0.1144300050000027	F	F	F
0.2500812949514900	0.7499156408597203	0.2753142807522539	T	T	T
0.4614599800000008	0.7885299619999984	0.1190500060000019	F	F	F
0.3766090120472632	0.8733798561568761	0.2783834447072922	T	T	T
0.2547900269999985	0.8771500269999990	0.0387400030000009	F	F	F
0.4228399730000021	0.9613300110000012	0.2022899959999975	F	F	F
0.4615100070000011	0.9224699410000028	0.1191200009999989	F	F	F
0.3765240458159545	0.7528610223663812	0.2784259842376225	T	T	T
0.3275099949999998	0.7884799970000032	0.1191200009999989	F	F	F
0.4969913895303260	0.8733714446739171	0.2784016945276654	T	T	T
0.2501599539999972	0.9998199560000032	0.0462899999999991	F	F	F
0.4167299260000021	0.8332699750000003	0.2062699860000023	F	F	F
0.3728499489999990	0.9952099540000034	0.0387400030000009	F	F	F
0.2886700179999977	0.8271600349999986	0.2022899959999975	F	F	F
0.3729199750000021	0.8770700239999982	0.0387100040000021	F	F	F
0.2887299710000022	0.9612699349999971	0.2023200000000003	F	F	F
0.3335199759999981	0.9164699929999998	0.1144300050000027	F	F	F
0.5001249429514892	0.7499484210090512	0.2752229761662270	T	T	T
0.7114599590000026	0.7885299619999984	0.1190500060000019	F	F	F
0.6266270110925914	0.8734255395159577	0.2783727788554402	T	T	T
0.5047900060000003	0.8771500269999990	0.0387400030000009	F	F	F
0.6728399940000003	0.9613300110000012	0.2022899959999975	F	F	F
0.7115099860000029	0.9224699410000028	0.1191200009999989	F	F	F
0.6265806055082211	0.7527518322447103	0.2783033856100932	T	T	T
0.5775100159999980	0.7884799970000032	0.1191200009999989	F	F	F
0.7470252248299156	0.8733794443869813	0.2784127210817821	T	T	T
0.5001699390000027	0.9998199560000032	0.0462899999999991	F	F	F
0.6667299470000003	0.8332699750000003	0.2062699860000023	F	F	F
0.6228499699999972	0.9952099540000034	0.0387400030000009	F	F	F
0.5386700179999977	0.8271600349999986	0.2022899959999975	F	F	F
0.6229199539999968	0.8770700239999982	0.0387100040000021	F	F	F
0.5387299920000004	0.9612699349999971	0.2023200000000003	F	F	F

0.5835199970000033	0.9164699929999998	0.1144300050000027	F	F	F
0.7500736394844746	0.7499152554712818	0.2752736385606200	T	T	T
0.9614599800000008	0.7885299619999984	0.1190500060000019	F	F	F
0.8766097543175538	0.8734256192977081	0.2783772785534104	T	T	T
0.7547899850000022	0.8771500269999990	0.0387400030000009	F	F	F
0.9228399730000021	0.9613300110000012	0.2022899959999975	F	F	F
0.9615100070000011	0.9224699410000028	0.1191200009999989	F	F	F
0.8767007374313875	0.7531138909889289	0.2784188956644500	T	T	T
0.8275100370000033	0.7884799970000032	0.1191200009999989	F	F	F
0.9970367377646764	0.8733969796601188	0.2784014314699316	T	T	T
0.7501699170000009	0.9998199560000032	0.0462899999999991	F	F	F
0.9167299260000021	0.8332699750000003	0.2062699860000023	F	F	F
0.8728499910000025	0.9952099540000034	0.0387400030000009	F	F	F
0.7886700390000030	0.8271600349999986	0.2022899959999975	F	F	F
0.8729199750000021	0.8770700239999982	0.0387100040000021	F	F	F
0.7887299710000022	0.9612699349999971	0.2023200000000003	F	F	F
0.8335200180000015	0.9164699929999998	0.1144300050000027	F	F	F
0.0000843823909149	0.7499054041235967	0.2753293973135386	T	T	T
0.4588600683813930	0.4461099175382134	0.3773146494560885	T	T	T
0.4092460351154487	0.5113070867813601	0.3872980478243437	T	T	T
0.5147780228820462	0.6220018591791010	0.3883666673800796	T	T	T
0.6185748447569209	0.6142828423318301	0.3848561088496122	T	T	T
0.6192497377805483	0.5116334426858343	0.3840961374283062	T	T	T
0.5113849074957165	0.5130282458981806	0.3799604807952531	T	T	T
0.4794645553516670	0.5529957290149590	0.3854936672195507	T	T	T
0.5833996234777168	0.5456665016401179	0.3820535011994063	T	T	T
0.3992126349788703	0.4487398854618879	0.3813863433536794	T	T	T
0.5828932820911226	0.6479566513186362	0.3862951581461043	T	T	T
0.4615146786695763	0.4058831800303919	0.3633101315883724	T	T	T
0.3496629339547486	0.4018641539851592	0.3834280893597338	T	T	T
0.6143954033102015	0.7038900157395351	0.3885409289127596	T	T	T
0.6691761626149961	0.5391955436725951	0.3740021695842081	T	T	T
0.5949932114823048	0.4611073876582398	0.3752451725331402	T	T	T

Data S3. CONTCAR of adenine adsorbed on C-111.
